# Early Sensory-Predominant Guillain-Barré Syndrome With Normal Cerebrospinal Fluid and MRI Findings: A Diagnostic Challenge

**DOI:** 10.7759/cureus.98602

**Published:** 2025-12-06

**Authors:** Salah Riyadh, Robert Knopp

**Affiliations:** 1 Emergency Department, Pikeville Medical Center, Pikeville, USA

**Keywords:** acute inflammatory demyelinating polyradiculoneuropathy, cerebrospinal fluid, diagnostic challenge, guillain‒barre syndrome, intravenous immunoglobulin, nerve conduction studies, peroneal neuropathy, sensory neuropathy

## Abstract

Guillain-Barré syndrome (GBS) is an acute, immune-mediated polyneuropathy that classically presents with progressive, symmetric weakness and areflexia following a preceding infection. However, early or atypical presentations may lack the hallmark diagnostic features, such as elevated cerebrospinal fluid (CSF) protein or abnormal magnetic resonance imaging (MRI) findings, creating a diagnostic challenge. We present the case of a 46-year-old woman who developed progressive distal paresthesias, gait instability, perioral numbness, hoarseness, and later right foot-drop following a viral-like illness. Initial evaluation revealed normal CSF protein levels, an unremarkable MRI of the brain and spine, and preserved strength, leading to diagnostic uncertainty. Despite these findings, the clinical pattern raised suspicion for early sensory-predominant GBS, and intravenous immunoglobulin therapy was initiated with subsequent clinical improvement. This case underscores the importance of maintaining high clinical suspicion for GBS in patients with compatible symptoms, even in the absence of classical diagnostic findings, to ensure timely intervention and prevent progression.

## Introduction

Guillain-Barré syndrome (GBS) is an acute, immune-mediated polyradiculoneuropathy that typically follows a viral or bacterial infection and is characterized by rapidly progressive, symmetric weakness and areflexia. The annual incidence ranges from one to two cases per 100,000 people and remains a leading cause of acute flaccid paralysis worldwide [1]. GBS encompasses several heterogeneous pathophysiologic subtypes, including acute inflammatory demyelinating polyradiculoneuropathy (AIDP), acute motor axonal neuropathy (AMAN), acute motor and sensory axonal neuropathy (AMSAN), and Miller Fisher syndrome. In North America, AIDP is the most prevalent subtype and is caused by immune-mediated demyelination of peripheral nerves and nerve roots. The classic diagnostic hallmark is albuminocytologic dissociation, elevated cerebrospinal fluid (CSF) protein without pleocytosis, along with demyelinating changes on nerve conduction studies [2].

However, atypical and early presentations can lack these characteristic findings, creating significant diagnostic uncertainty. Sensory-predominant variants, for instance, may present with distal paresthesias, pain, or gait disturbance before weakness develops, sometimes mimicking peripheral mononeuropathies or radiculopathies [3]. In such cases, both CSF and magnetic resonance imaging (MRI) may appear normal early in the disease course, and electrodiagnostic abnormalities may not yet be evident.

Early recognition remains essential, as prompt initiation of intravenous immunoglobulin (IVIG) or plasma exchange can reduce neurological progression and long-term disability. We present a case of early sensory-predominant GBS with normal CSF and MRI findings, highlighting the importance of maintaining clinical suspicion even when initial investigations are unrevealing.

## Case presentation

A 46-year-old woman with a history of anxiety, depression, and hypothyroidism presented to the emergency department with a 10-day history of flu-like symptoms, followed by progressive distal paresthesias, perioral numbness, hoarseness, and gait instability. She denied recent trauma, bowel or bladder dysfunction, dysphagia, or visual disturbances. Initial neurological examination demonstrated intact cranial nerves, preserved muscle strength in all extremities, and normal deep tendon reflexes. Sensory testing revealed diminished pinprick and vibration sensation in the distal lower extremities extending to the ankles.

**Figure 1:**
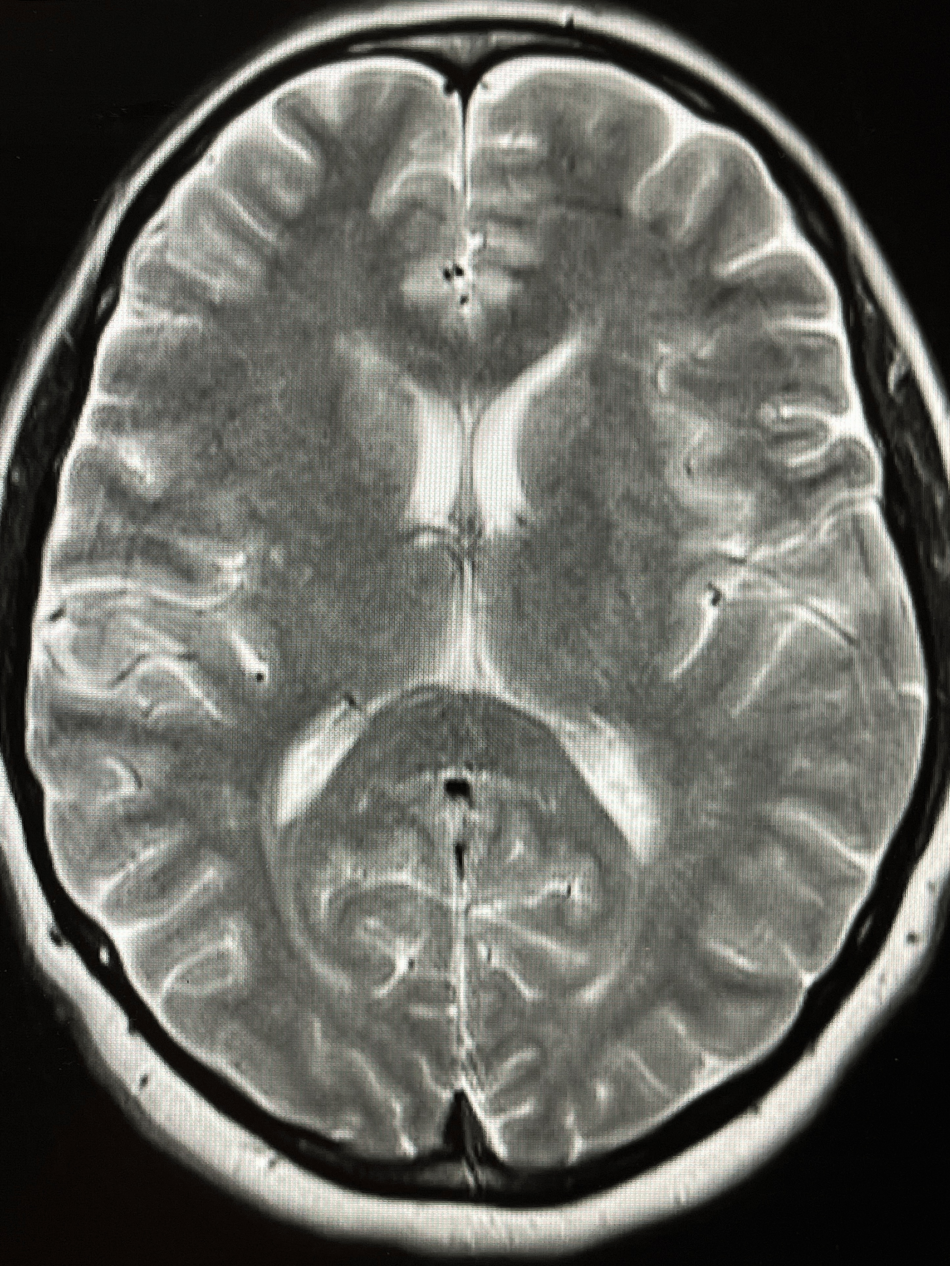
Axial T2-weighted MRI of the brain Axial T2-weighted magnetic resonance imaging (MRI) showing normal ventricular configuration and parenchymal signal without white-matter lesions or acute pathology.

**Figure 2:**
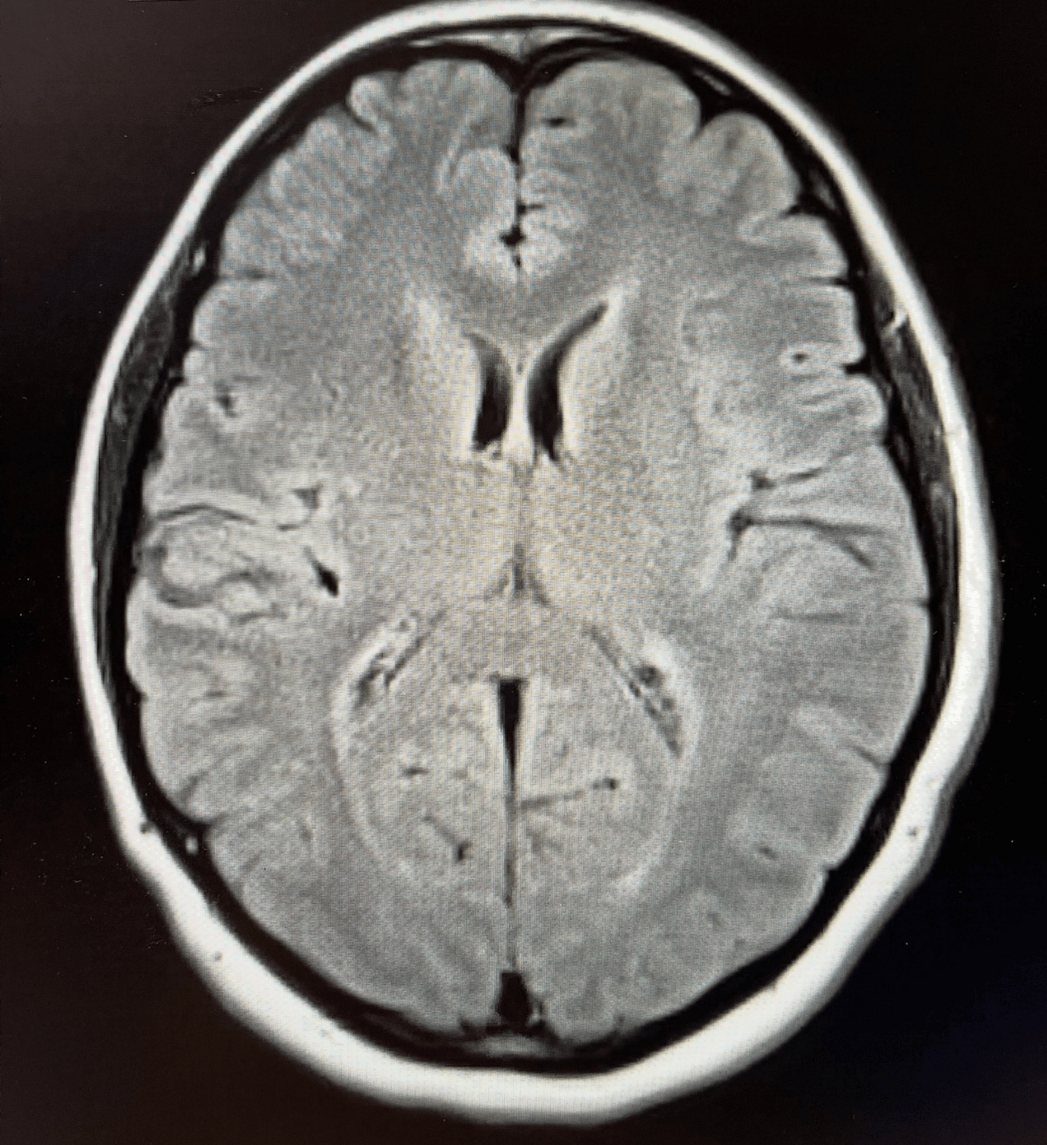
Axial FLAIR MRI of the brain Axial fluid-attenuated inversion recovery magnetic resonance imaging (FLAIR MRI) demonstrating no acute intracranial abnormalities, no white-matter lesions, and no imaging evidence of demyelinating disease such as multiple sclerosis.

Given the patient’s progressive sensory symptoms and otherwise unremarkable neurological examination, an initial diagnostic evaluation was pursued. Magnetic resonance imaging (MRI) of the brain (Figures 1-2) was ordered and demonstrated no acute intracranial abnormalities and no evidence of demyelinating disease.

**Figure 3:**
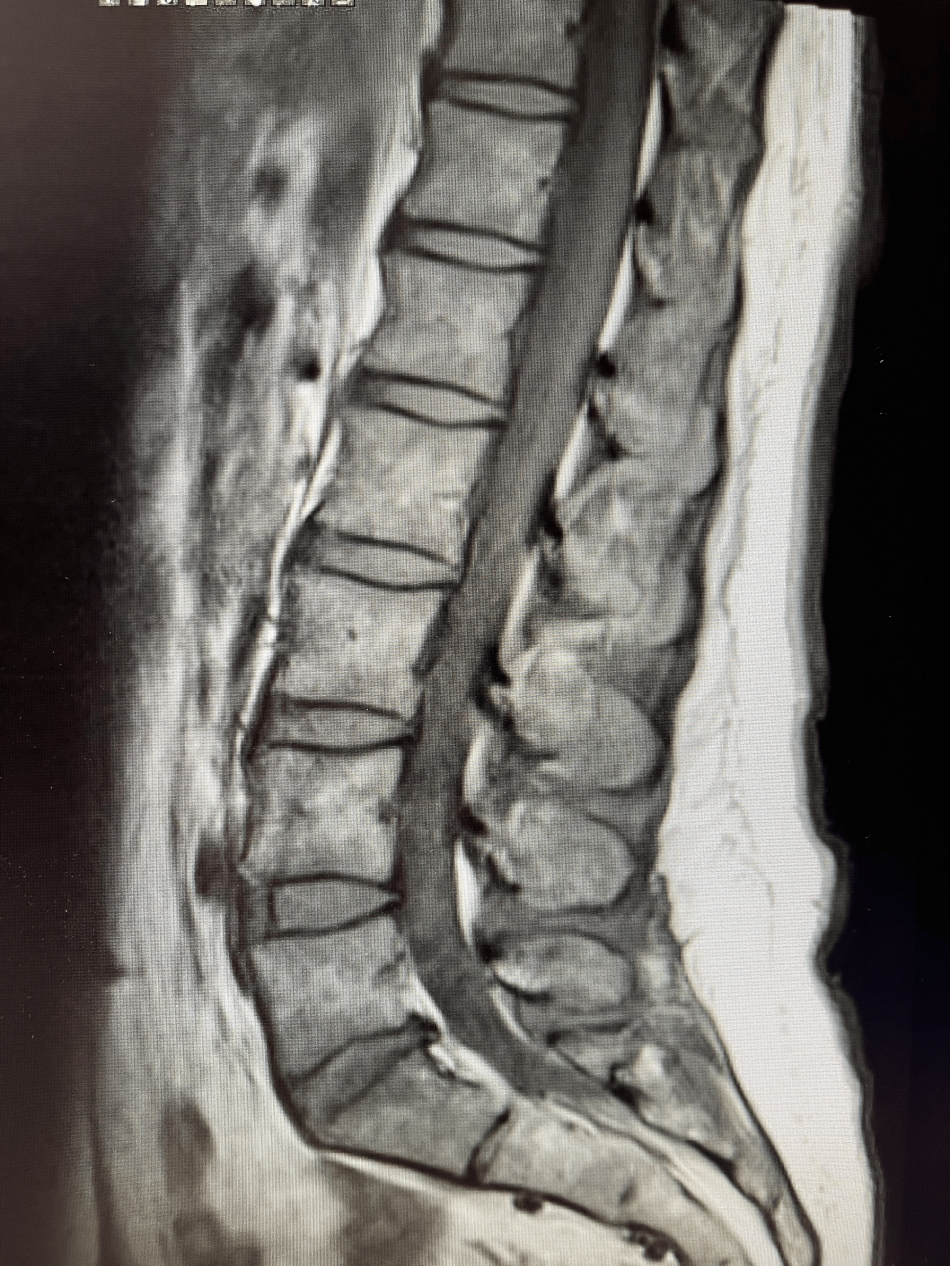
Sagittal T2-weighted MRI of the lumbar spine Sagittal T2-weighted magnetic resonance imaging (MRI) of the lumbar spine demonstrating multilevel degenerative disc and facet joint disease with mild spinal canal stenosis, without evidence of cauda equina inflammation, nerve-root enhancement, or acute demyelinating pathology.

MRI of the lumbar spine (Figure 3) was also obtained to further evaluate for spinal pathology and revealed multilevel degenerative disc and facet joint disease with mild canal stenosis, without cauda equina inflammation or nerve-root enhancement to suggest an inflammatory polyradiculoneuropathy.

CSF obtained via lumbar puncture was clear and colorless with normal glucose (54 mg/dL) and protein (27 mg/dL). Red blood cells measured 38 cells/µL and nucleated cells measured 120 cells/µL, without evidence of infection. There was no albuminocytologic dissociation. The CSF results are summarized in Table 1.

**Table 1:** Cerebrospinal fluid (CSF) analysis CSF obtained via lumbar puncture demonstrating normal glucose and protein levels, with clear and colorless appearance. Mildly elevated red blood cells and nucleated cells were present, likely attributable to a traumatic tap. No albuminocytologic dissociation was observed.

Parameter	Patient value	Reference range
Appearance (clarity)	Clear	Clear
Color	Colorless	Colorless
Glucose, CSF	54 mg/dL	40-70 mg/dL
Protein, CSF	27 mg/dL	15-45 mg/dL
Red blood cells (RBC), CSF	38 cells/µL	0-5 cells/µL
Total nucleated cells (WBC), CSF	120 cells/µL	0-5 cells/µL

Laboratory evaluation, including complete blood count (CBC), comprehensive metabolic panel (CMP), thyroid-stimulating hormone, erythrocyte sedimentation rate, C-reactive protein, and procalcitonin, was within normal limits. Complete laboratory data are summarized in Tables 2-4.

**Table 2:** Comprehensive metabolic panel Comprehensive metabolic panel showing normal electrolyte balance, renal function, liver enzymes, and serum proteins. No abnormalities were identified apart from a mildly elevated albumin-to-globulin ratio, which was not clinically significant in this context. A/G: albumin/globulin, ALT: alanine transaminase, AST: aspartate transaminase, BUN: blood urea nitrogen, eGFR: estimated glomerular filtration rate

Parameter	Patient value	Reference range
A/G ratio	1.7	0.6-1.6
Albumin	4.3 g/dL	3.5-5.1 g/dL
Alkaline phosphatase	61 U/L	29-108 U/L
ALT	15 U/L	<40 U/L
Anion gap	6 mmol/L	5-15 mmol/L
AST	13 U/L	10-28 U/L
BUN	12 mg/dL	7-20 mg/dL
BUN/creatinine ratio	14.81	10.0-20.0
Calcium	9.1 mg/dL	7.9-11.1 mg/dL
Chloride	106 mmol/L	99-108 mmol/L
CO2 (total)	27 mmol/L	19-29 mmol/L
Creatinine	0.81 mg/dL	<1.20 mg/dL
eGFR	87.4 mL/min/1.73 m²	>60-200 mL/min/1.73 m²
Globulin (total)	2.59 g/dL	2.4-4.8 g/dL
Glucose	81 mg/dL	58-104 mg/dL
Magnesium	2.2 mg/dL	1.8-2.4 mg/dL
Potassium	4.1 mmol/L	3.2-4.6 mmol/L
Sodium	139 mmol/L	134-143 mmol/L
Total bilirubin	0.5 mg/dL	0.3-1.0 mg/dL
Total protein	6.8 g/dL	5.9-7.9 g/dL

**Table 3:** Complete blood count (CBC) CBC results demonstrating normal white blood cell count, hemoglobin, hematocrit, red blood cell indices, and platelet parameters. No leukocytosis, anemia, thrombocytopenia, or abnormalities in red cell distribution were identified.

Parameter	Patient value	Reference range
White blood cell count (WBC)	9.91 x10³/µL	3.8-11.0 x10³/µL
Hemoglobin (Hgb)	14.7 g/dL	11.2-15.3 g/dL
Hematocrit (Hct)	43.2%	32.6-44.6%
Red blood cell count (RBC)	4.80 x10^6^/µL	3.73-5.13 x10^6^/µL
Mean corpuscular volume (MCV)	90.1 fL	78.8-96.0 fL
Mean corpuscular hemoglobin (MCH)	30.7 pg	26.2-33.0 pg
Mean corpuscular hemoglobin concentration (MCHC)	34.1 g/dL	32.7-35.1 g/dL
Red cell distribution width (RDW)	13.7%	12.1-16.1%
Platelet count	237 x10³/µL	138-402 x10³/µL
Mean platelet volume (MPV)	8.0 fL	7.0 -10.6 fL

**Table 4:** Additional diagnostic studies Additional diagnostic testing, including inflammatory markers and thyroid studies, demonstrated normal C-reactive protein, erythrocyte sedimentation rate, and procalcitonin levels, indicating no systemic inflammation or bacterial infection. Thyroid-stimulating hormone was within normal limits, making thyroid dysfunction an unlikely contributor to the patient’s symptoms.

Parameter	Patient value	Reference range
C-reactive protein (CRP)	0.4 mg/dL	0.0-1.0 mg/dL
Procalcitonin	<0.01 ng/mL	0.01-0.50 ng/mL
Thyroid-stimulating hormone (TSH)	1.063 IU/mL	0.450-5.330 IU/mL
Erythrocyte sedimentation rate (ESR)	9 mm/hr	0-20 mm/hr

Given the progressive, symmetric, sensory-predominant pattern following a viral-like illness, the attending physician suspected early AIDP. Intravenous immunoglobulin (IVIG) therapy was initiated at 0.4 g/kg/day. The patient reported near-complete improvement in paresthesias and gait after two doses but declined the remaining treatments due to subjective improvement. She was discharged in stable condition with instructions for close outpatient follow-up.

At a two-week neurology visit, the patient noted improvement in upper extremity symptoms but worsening lower extremity weakness and right foot drop. Examination revealed absent patellar and Achilles reflexes, decreased pinprick sensation in the L4-L5 dermatomes, and 0/5 dorsiflexion strength on the right with 3/5 plantarflexion bilaterally. Findings were consistent with bilateral peroneal neuropathy superimposed on a demyelinating process. Pregabalin 25 mg twice daily was prescribed, and she was referred to a neuromuscular specialist for further evaluation.

This case demonstrates an atypical sensory-predominant presentation of early Guillain-Barré syndrome, with initially normal brain MRI findings (Figures 1-2) and nondiagnostic CSF analysis (Table 1).

## Discussion

GBS is classically characterized by rapidly progressive, symmetric weakness, diminished reflexes, and sensory disturbances following an antecedent infection. In North America, the most common subtype is AIDP, an immune-mediated process in which molecular mimicry between infectious antigens and peripheral nerve components triggers inflammatory demyelination of nerve roots and peripheral nerves [1,2]. Although motor deficits predominate in typical presentations, a spectrum of clinical variants has been increasingly recognized, including sensory-predominant forms, localized neuropathies, and cases with minimal or delayed weakness [3,4].

Sensory-predominant variants may initially manifest with distal paresthesias, neuropathic pain, or gait instability, and can closely mimic peripheral neuropathies or radiculopathies. These atypical presentations contribute to diagnostic uncertainty, particularly early in the disease course when classical features have not yet evolved. Albuminocytologic dissociation-elevated CSF protein with normal cell counts is a key diagnostic feature but may not be present until one to two weeks after symptom onset, making early lumbar puncture nondiagnostic in a substantial proportion of patients [2,5]. This was consistent with our patient, whose CSF analysis showed normal protein and glucose without pleocytosis, as summarized in Table 1. Serologic testing for anti-ganglioside antibodies was not obtained, as these assays are not available emergently in the emergency department and do not influence acute management decisions; moreover, negative or absent antibody results do not exclude early AIDP, as antibody positivity varies across GBS subtypes.

Neuroimaging is typically normal in GBS and primarily serves to exclude alternative diagnoses. In our patient, brain MRI (Figures 1, 2) showed no demyelinating lesions or acute intracranial abnormalities, and lumbar spine MRI (Figure 3) demonstrated degenerative changes without cauda equina inflammation or nerve-root enhancement. While enhancement of the cauda equina or nerve roots may be observed in more advanced cases, the absence of such findings does not exclude AIDP [1,2]. Electrodiagnostic studies are often considered the most informative diagnostic tool for confirming demyelination; however, characteristic abnormalities may not appear until several days after symptom onset, and early studies can be normal or equivocal [3,6]. Nerve conduction studies and EMG were not obtained during the patient’s initial evaluation because she presented to the emergency department, where electrodiagnostic testing is not readily available and is not routinely performed during early symptom onset. In addition, early NCS findings may be normal or equivocal, limiting their diagnostic utility in the acute setting.

Timely initiation of immunotherapy remains essential, as both intravenous immunoglobulin (IVIG) and plasma exchange have demonstrated efficacy in reducing disease progression, respiratory failure, and long-term disability [7,8]. Our patient demonstrated partial improvement in sensory symptoms following IVIG initiation despite nondiagnostic CSF and MRI findings, supporting early immune involvement. Her serum laboratory studies, including CBC, CMP, inflammatory markers, and thyroid studies (Tables 2-4) were unremarkable, further highlighting the challenge of early diagnosis in atypical presentations. The subsequent development of bilateral peroneal neuropathy and right foot drop likely represented the progression of the underlying inflammatory process rather than an isolated compressive neuropathy.

This case underscores several important clinical considerations. First, GBS should remain a key differential diagnosis in patients presenting with progressive sensory symptoms following a viral-like illness, even when early diagnostic studies, including CSF (Table 1) and MRI (Figures 1-3), are normal. Second, reliance on early CSF or MRI findings alone may delay diagnosis; clinicians must integrate clinical progression and pattern recognition. Finally, early treatment and close follow-up are critical, as delayed motor involvement or new deficits may emerge despite initial improvement.

## Conclusions

Early or sensory-predominant variants of GBS may present with subtle findings and normal diagnostic studies, creating challenges in recognition and management. Clinicians should maintain a high index of suspicion for GBS in patients with progressive, symmetric sensory symptoms following a viral-like illness, even when CSF and MRI results are unrevealing. Early initiation of immunotherapy can prevent disease progression and improve outcomes. This case emphasizes the importance of prompt medical assessment, close follow-up, and clinical vigilance in patients with atypical or evolving presentations of GBS.
